# Dr. Ferguson on Auscultation in Pregnancy

**Published:** 1831-04-01

**Authors:** 


### Periscope;

OR,

### CIRCUMSPECTIVE REVIEW.

"Ore trahit quodcunque potest, atque addit acervo."

### Auscultation the only Unequivocal Evidence of Pregnancy. By Dr. Ferguson, A.M. M.B., Dublin.*

Such is the rather equivocal title of a paper of some length in the Dublin Medical Transactions. We all know that the signs of pregnancy, though usually distinct, are occasionally doubtful, if not absolutely fallacious. There are few medical men of any practice, who have not been at one time or other deceived. The discovery of an infallible, or certain sign is confessedly a desideratum. Many of our readers are aware no doubt that Laennec alludes to the employment of the stethoscope in the detection of pregnancy, and some will probably smile on the occasion. The signs detected by auscultation are two; the 'bruit placentaire,' and the pulsations of the fœtal heart. They are seldom observed before the fifth month, and seldom fail to be recognized by an accurate observer after it. The 'bruit placentaire' should be sought for in either iliac region, though Dr. Ferguson avers that he has found it in almost every point of the abdomen. He thinks it is nothing more than the bruit de soufflet in the arteries connecting the placenta to the uterus. The Doctor has heard the fœtal heart in almost every region of the abdomen. Though it and the placenta are sometimes heard on the same side, and have even been found on the same spot, yet in the majority of cases they are met with at opposite sides, generally in the iliac region. The fœtal heart, unlike the placenta, is not always heard in the same place in the same individual, that is, it may be found to-day in one point, to-morrow in another, though Dr. F. has never observed it to vary far from the point where first heard. Its double beat is well marked, and the frequency of its pulsations almost always greater, often double that of the mother's heart. In one case, however, the fœtal heart was distinctly heard to beat only 28, the mother's being 100, and there was an irregularity in its rhythm. The following is a curious instance of that talent for minute stethoscopic observation which appears to characterise Dr. Corrigan and some others in Dublin.

"A goat had been procured for a very different purpose by Doctors Hunt, Corrigan and myself, and bound on its back on the operating table. I casually applied the stethoscope to its abdomen, without the slightest previous knowledge of its pregnancy, and was surprised to detect almost immediately the distinct double pulsations of a fœtal heart. My two friends, to whose accuracy of observation I have been often indebted, satisfied themselves perfectly of the fact, and on examining the uterus about an hour afterwards, we extracted a fœtus, of which the minute preparation which I now offer was the heart. On enquiring from the person who sold us the goat, on whose accuracy we could depend, we learned that it was exactly seven weeks from copulation."

It requires nice ears, we should opine, to count with the precision of a pendulum the double pulsations of a fœtal heart, "the size of a hazel nut," in the belly of a goat bound upon a table. We

* Dublin Med. Transactions, Vol. I, Part I.

quite envy Drs. Corrigan, Hunt, and Ferguson. With the following statement, which deserves attention, we proceed to one of Dr. Ferguson's cases.

"I would beg leave to state, that for the last three years I have been in the habit of examining by the stethoscope every pregnant woman who presented herself, under any circumstances, at either of the public Institutions to which I am attached. I have thus had opportunities of testing the value of mediate auscultation in such cases above one hundred times, and in every instance, with but one exception, I could detect either pulsation of the fœtal heart or placentary noise, generally both, after the patient had passed the fifth month of gestation, and in many, indeed the majority, before that period. In the individual case which I have excepted, I could detect neither the one nor the other; however, I had no opportunity of making a second examination, which should never be omitted in doubtful cases, nor of learning whether the child might have been dead or not; for the woman, contrary to her promise, never returned to me. I should also remark, that in not more than eight or ten cases did I find it necessary to remove the usual clothes of the patient from beneath the instrument, leaving only the chemise between it and the abdominal parietes; nor to oblige them to assume the horizontal position, which is decidedly more favorable to an accurate examination than the erect or sitting. My usual practice has been to examine them seated on a chair, and without the removal of any part of their dress. *I am well aware that others, who may have made frequent trials, have not been so fortunate in detecting these phenomena:* this is not for me to attempt to explain nor contend about; my sole object is to state facts which I have myself observed, and only claim, what I hope I shall obtain, credit at least for veracity."

*Case.* In December, 1828, Dr. Ferguson met an eminent general practitioner in consultation, on the case of the daughter of a respectable shopkeeper. Her apparent disease was ascites. She stated that her ailments had been of twelve months standing; that during that time her abdomen had been occasionally enlarged, though more permanently so of late; and that remedies had produced the most beneficial effects, all of which was confirmed by her mother. The agitated manner of the young woman, and the peculiar shape and solidity of the belly roused Dr. Ferguson's suspicion. Not having a stethoscope with him, he used a roll of paper, and with much difficulty and embarrassment succeeded in discovering the pulsations of the fœtal heart. The announcement of the diagnosis surprised the practitioner, who continued incredulous till an accouchement dispelled all doubts.

We need not pursue the subject farther, though our readers might be pleased with samples of the eloquence displayed on the occasion by Dr. Ferguson. We may here make an observation which has struck us forcibly on perusing more than one paper in the present Transactions of the Dublin physicians. The verbiage in almost all the memoirs is astonishingly great, and never did we witness facts more buried beneath it. If the case of a young lady is described, we are let into some of the family history, informed that the father is of an economical or liberal turn, that the Doctor got or did not get fees. In Dr. Ferguson's memoir, the cases are recited in a tragic or comic style, and a fact that might have laid in as small a compass as the heart of the fœtal kid, is dilated and diluted into some goodly octavo pages. We must also remark that, in these Transactions, a vast deal of eloquence is thrown away upon nothing. If a stool is to be described, it is done in language more florid than the most bilious evacuation ever procured by calomel. We know that this generous warmth of expression is said to be more peculiarly characteristic of our countrymen of the Emerald Isle; but however delightful it may be in works of imagination, or the harangues of the lawyer and the senator, we conceive that it is out of place in sober discussions. We trust that this hint will be taken in good part.

